# Altered DNA Methylation Pattern Contributes to Differential Epigenetic Immune Signaling in the Upper Respiratory Airway of Unvaccinated COVID-19 Patients

**DOI:** 10.3390/cells14211673

**Published:** 2025-10-27

**Authors:** Melissa Govender, Jyotirmoy Das, Francis R. Hopkins, Cecilia Svanberg, Johan Nordgren, Marie Hagbom, Jonas Klingström, Åsa Nilsdotter-Augustinsson, Yean K. Yong, Vijayakumar Velu, Sivadoss Raju, Johanna Sjöwall, Esaki M. Shankar, Sofia Nyström, Marie Larsson

**Affiliations:** 1 Division of Molecular Medicine and Virology, Department of Biomedical and Clinical Sciences, Linköping University, 581 83 Linköping, Sweden; francis.hopkins@liu.se (F.R.H.); cecilia.svanberg@liu.se (C.S.); johan.nordgren@liu.se (J.N.); marie.hagbom@liu.se (M.H.); jonas.klingstrom@liu.se (J.K.); sofia.c.nystrom@liu.se (S.N.); 2 Centre for Human Genetics, Pandemic Sciences Institute, University of Oxford, Oxford OX3 7BN, UK; 3 Bioinformatics, Core Facility, Division of Cell Biology, Department of Biomedical and Clinical Sciences, Faculty of Medicine and Health Sciences, Linköping University, 581 85 Linköping, Sweden; jyotirmoy.das@liu.se; 4 Clinical Genomics Linköping, SciLife Laboratory, Department of Biomedical and Clinical Sciences, Linköping University, 581 83 Linköping, Sweden; 5 Division of Infection and Inflammation, Department of Biomedical and Clinical Sciences, Linköping University, 581 83 Linköping, Sweden; asa.nilsdotter-augustinsson@liu.se (Å.N.-A.); johanna.sjowall@liu.se (J.S.); 6 Department of Infectious Diseases, Vrinnevi Hospital, 603 79 Norrköping, Sweden; 7 Laboratory Centre, Xiamen University Malaysia, Sepang 43900, Selangor, Malaysia; ykyong@xmu.edu.my; 8 Laboratory Medicine, Department of Pathology, School of Medicine, Emory University, Atlanta, GA 30322, USA; vvelu@emory.edu; 9 Division of Microbiology and Immunology, Emory Vaccine Center, Emory National Primate Research Center, Emory University, Atlanta, GA 30329, USA; 10 State Public Health Laboratory, Directorate of Public Health and Preventive Medicine, DMS Campus, Teynampet, Chennai 600 018, Tamil Nadu, India; sivraju@gmail.com; 11 Infection and Inflammation, Department of Biotechnology, Central University of Tamil Nadu, Thiruvarur 610 005, Tamil Nadu, India; shankarem@cutn.ac.in; 12 Department of Clinical Immunology and Transfusion Medicine, Linköping University, 583 30 Linköping, Sweden

**Keywords:** DNA methylation, COVID-19 patients, airway, nasopharyngeal, epigenetic, unvaccinated COVID-19 patients

## Abstract

**Highlights:**

**What are the main findings?**

- COVID-19 patients show a unique DNA methylation profile in the upper airway, with over 510,000 differentially methylated CpGs affecting antiviral, interferon, and immune response genes.
- Some methylation changes are temporary, normalizing after 6 weeks, while key immune regulators (e.g., IL17A, ERK1/2, OAS1, MX1) remain significantly involved.

**What is the implication of the main finding?**

- SARS-CoV-2 may reprogram immune and repair pathways in the airways, influencing recovery and susceptibility to future respiratory infections.
- These findings provide potential targets for biomarkers and therapeutic strategies to modulate post-COVID-19 airway health.

**Abstract:**

SARS-CoV-2 infection remains a global health concern, with its impact on host immune responses not fully understood. In a case–control study, we examined how COVID-19 affects DNA methylation patterns in the upper respiratory airway of hospitalized individuals. DNA methylation arrays were performed on nasopharyngeal samples at inclusion/hospitalization and 6 weeks post-inclusion. We found a distinct DNA methylation pattern in COVID-19 patients compared to healthy controls, identifying 510,099 differentially methylated CpGs. Within the transcription start sites (TSSs) and gene body, COVID-19 patients displayed a higher number of genes/CpGs with elevated methylation levels. Enrichment analysis of TSS-methylated genes revealed effects of SARS-CoV-2 on genes associated with type I interferons, anti-viral and inflammatory responses, and immune functions. Some CpG methylations were transient, and normalized at group level by 6 weeks post-inclusion. Several IFN-regulated genes, including OAS1, OAS3, IFIT3, and MX1, were identified. Among the top regulators were IL17A and ERK1/2, both involved in inflammatory processes. Networks nodes included IGF1 and EGF, associated with processes including tissue repair and activation of immune responses. Overall, our data suggests that COVID-19 can impact the upper airway by modifying gene methylation patterns. This could have implications for conditioning of the airways, how individuals respond to future airway infections, and therapeutic interventions.

## 1. Introduction

The rapid spread and consistent evolution of SARS-CoV-2 variants signifies that this virus continues to impact human health and society. Since the onset of the COVID-19 pandemic in 2020, substantial advances have been achieved in therapeutics, together with the development of a wide array of vaccines to combat the pandemic [1]. Despite increased control measures the world has witnessed a high death toll due to SARS-CoV-2 infection, largely among non-vaccinated individuals [2]. Furthermore, the diverse spectrum of symptoms and the long-lasting impact among people with severe COVID-19 [3,4] together with the viral evolution and risk of emergence of new SARS-CoV-2 variants of concern [5,6] continue to pose challenges to effective disease prevention. The severity of COVID-19 has been linked to dysregulation of immune responses induced by exaggerated and prolonged inflammation [7]. Hence, a thorough understanding of the nature of immune responses elicited against SARS-CoV-2 is of paramount importance and relevance.

A large proportion of COVID-19 research has been devoted to phenotype, proteomic [8,9], and/or transcriptomic studies [10,11,12], which so far have given vital knowledge on the dynamics of the immune cell landscape. In a previous study, we revealed persistent alterations within the monocytic, dendritic cell, and T cell compartments induced by SARS-CoV-2 infection, which still remained six to seven months post-hospitalization [13,14]. This underscores the enduring impact of SARS-CoV-2 on immune cell dynamics, highlighting the necessity for continued exploration into the intricacies of immune responses to the virus.

More recently, there have been multiple epigenetic studies aimed at unraveling the complexities of SARS-CoV-2 pathogenesis and its impact on the host [15,16,17]. Of the various epigenetic modifications, DNA alterations, and particularly methylation, exhibit remarkable stability and form an integral component of the programming of a cell, persisting throughout its life cycle and divisions. The activation or silencing of certain genes by DNA modifications is one of the immune regulatory mechanisms implicated in the control of infection and the attrition of inflammatory responses to avoid tissue damage in the host [18]. The regulatory influence of DNA methylation extends to transcriptional accessibility of genes and the subsequent effects, i.e., gene activity and expression, depending on the region in the genome/gene affected [19,20]. Many biological and environmental factors appear to influence the DNA methylation patterns such as age, biological sex, and body mass index (BMI) [21,22]. Likewise, viral and bacterial infections can elicit rapid epigenetic alterations, including DNA methylation, and thereby regulate gene expression in cells [5,6,23].

Blood cells DNA methylation patterns in people with subclinical or mild COVID-19 differed from patterns found in healthy individuals, with enrichment of, e.g., muscarinic acetylcholine receptor signaling and gonadotropin-releasing hormone receptor pathways, in patients with COVID-19 [24]. Unique SARS-CoV-2-specific methylation patterns were also found in COVID-19 patients compared to pre-pandemic healthy controls and to patients with rhinovirus and influenza B virus upper respiratory infections [25]. In addition, the immune functions in SARS-CoV-2-infected individuals are also affected by DNA methylation, and certain methylation profiles in peripheral blood samples have been linked to disease severity, such as respiratory distress and intensive care unit (ICU) admission [25,26].

COVID-19 patients with acute respiratory distress syndrome (ARDS) had 14% differentially methylated genes in the promoter regions compared to healthy controls and they are involved in regulating immune pathways, such as IFN-γ and IFN-α [27]. In addition, hypermethylation of genes in the ‘apoptotic execution pathway’ has been linked to increased risk of mortality in COVID-19 patients [27]. Furthermore, several DNA methylations occurred in the absent in melanoma (AIM) inflammasome gene during COVID-19 progression, which were linked to heightened immune responses among patients who recovered from SARS-CoV-2 infection [27].

Several epigenetic studies have investigated the methylation patterns in blood samples of individuals with mild to severe COVID-19 [24,25,26,27]. SARS-CoV-2 infection is however initiated in the upper airways, establishing a microenvironment involving the respiratory airway mucosa and immune cells of the airway mucosa that should considerably differ from those distributed in the blood. In this study we thus aimed to explore the methylation status in the upper respiratory airway using nasopharyngeal specimens obtained from hospitalized patients with moderate to severe COVID-19. We observed significant differences in the methylation patterns among COVID-19 patients at hospitalization/inclusion and at 6 weeks post-inclusion relative to healthy controls. Enrichment analyses of methylated genes at transcription start sites in COVID-19 patients highlighted the impact on inflammatory responses and immune processes. Certain SARS-CoV-2-induced CpG methylations were transient, reverting to normal levels within 6 weeks post-hospital admission. Notably, the pro-inflammatory factor IL17A and signaling factor ERK1/2 were among the top regulators affected by the infection. In addition, IGF1 and EGF were central nodes and are both factors that influence various biological processes in COVID-19.

## 2. Materials and Methods

### 2.1. Participants and Sample Collection

The study was approved by the Swedish Ethical Review Authority (Ethics No. 2020-02580) and was carried out in accordance with the Declaration of Helsinki. The patient cohort included unvaccinated, hospitalized COVID-19 patients (N = 27; age range 26–91 years), and SARS-CoV-2-negative, unvaccinated, healthcare workers as healthy controls (HC) (N = 12; age range 26–62 years), from our previously reported cohort [13,14]. All individuals provided written informed consent prior to enrolment. The COVID-19 patient clinicodemographic data are described in Table 1.

COVID-19 disease score was defined according to the criteria advocated by the National Institutes of Health [4,28] and slightly modified based on the requirement for supplementary oxygen and the highest level of care required. Based on the above criteria, the disease status of patients in this cohort were classified mostly as moderate to severe (Table 1). Nasopharyngeal specimens were collected at inclusion, i.e., timepoint (T)1 and at 6 weeks post-inclusion, i.e., timepoint (T)2. The COVID-19 patient cohort comprised of nine pairs from volunteers (i.e., T1 and T2), twelve unpaired T1 volunteers, and six unpaired T2 samples. The samples were transferred into microfuge tubes and centrifuged at 1800 RPM for 6 min at 4 °C, and the pellets were frozen at −80 °C for later extraction of genomic DNA (gDNA) and analysis.

### 2.2. Genomic DNA Isolation

Genomic DNA (gDNA) was isolated using a Qiagen AllPrep DNA/RNA Mini kit (Qiagen, Hilden, Germany) according to the manufacturer’s instructions. Briefly, 600 µL of lysis buffer (RLY) was added to the thawed pellets and resuspended. The lysate was transferred to an AllPrep DNA column and centrifuged at 11,000× *g* for 1 min. The column was washed with 500 µL AW1 buffer (centrifuged at 11,000× *g* for 1 min) and washed with 500 µL AW2 buffer. Finally, the columns were placed in 1.5 mL microfuge tubes, and 30 µL extraction buffer (EB) was added for a 1 min incubation before eluting the gDNA by centrifuging at 11,000× *g* for 1 min. The elution step was repeated once with the eluant to increase the gDNA concentration.

### 2.3. Quantification of Genomic DNA

The quality and concentration of the genomic DNA was measured using a broad range (BR) dsDNA Quantitation Qubit^TM^ assay (Invitrogen, Waltham, MA, USA) according to the manufacturer’s instructions. Briefly, the samples and standards were diluted in the Qubit working solution (Qubit reagent diluted 1:200 in Qubit buffer) and vortexed for a few seconds before incubation for 2 min at room temperature. The amount of genomic DNA was then quantified by measuring the fluorescence with a Qubit^TM^ 2.0 Fluorometer (Invitrogen^TM^ by Life Technologies^TM^, Thermofisher, Waltham, MA, USA).

### 2.4. Sample Preparation for DNA Methylation Microarray

Quantified gDNA samples (69–552 ng) were transported to the Bioinformatics and Expression Analysis Core facility (BEA), Karolinska Institute, Stockholm, Sweden. There, the samples were subjected to bisulfite conversion using a Zymo Research EZ-96 DNA Methylation™ Kit (D5004) (Nordic Biosite, Täby, Sweden). The samples were subsequently quantified with a Qubit assay, and 70–250 ng gDNA was used as input for the Infinium MethylationEPIC v1.0 BeadChip (Illumina Inc., San Diego, CA, USA) following the protocol outlined by Illumina. QC, normalized data, and IDAT files were obtained and used for further analysis.

### 2.5. Data Processing and Statistical Analysis: Differential Methylation Analysis

The IDAT files from Illumina^®^ HumanMethylation EPIC arrays (San Diego, CA, USA) were analyzed using R (v4.3.1) [29] and Bioconductor packages (v3.16). The Chip Analysis Methylation Pipeline (ChAMP) [30] analysis package (v2.28.0) was used for initial analysis. The data were pre-processed with the default ChAMP filter option to filter out the following: (1) CpGs with detection *p* value > 0.01 (removing 32,515 probes); (2) bead count of <3 (removing 5192 probes); (3) all non CpG probes (removing 2760 probes); (4) all SNP-related probes as identified in Zhou’s Nucleic acids Research article 2016 [31] (removing 94,414 probes); (5) probes that align to multiple locations, as identified in Nordlund et al. [32] (removing 7182 probes); (6) probes located on X and Y chromosome (removing 15,616 probes). This resulted in 715,410 probes. The filtered data was normalized using the beta-mixture quantile normalization (BMIQ) function using the ChAMP package [33].

We adjusted for variables such as batch effects using the surrogate variable analysis (SVA) package (v3.54.0) and the principal component regression (PCR) analysis function using EnMix package (v1.38.1) for all variables, slide (batch), age, biological sex, C-reactive protein (CRP), lactate dehydrogenase (LDH), body mass index (BMI), and smoking status, against the sample group using the following equation:

$${data}_{sva}=sva(data,mod,mod0,n.sv)$$

where data is the transformed data matrix, mod it the model matrix being used to fit the data, mod0 is the null model, and n.sv is the number of surrogate variables to estimate (here in our case, n.sv = 0).

To estimate the cell-type composition in the data set we used meth_atlas (https://github.com/nloyfer/meth_atlas, accessed on 17 October 2025), which is a reference-based method to adjust cell-type composition in genome-wide DNA methylation studies from the heterogenous tissues [34]. The differential methylation analysis was estimated on the batch and cell-type corrected data using the linear modeling (lmFit) and eBayes algorithm between two sample groups. The differentially methylated CpGs were adjusted for multiple comparisons using a Bonferroni–Hochberg (BH)-corrected *p* value (*p*-value_BH_) < 0.05. The hierarchical cluster analysis was performed using the Euclidean distance calculation within the ape package [35] (v5.7). The principal component analysis was performed using FactoMineR [36] (v2.8) and factoExtra [37] (v1.0.7) packages. To estimate the genomic inflation, the Bacon package (v1.30.0) was used (with median inflation factor, λ = ~0.32, and average bias ~0.5). See Supplementary Figure S1 for control analysis, corrections, and calibrations of the data.

### 2.6. Downstream Analysis: Feature Analysis of Differentially Methylated CpGs

The resulting significant differentially methylated CpGs were annotated using Illumina manifest (Human Genome version 38). The ggplot package was used to visualize the genomic distribution. The volcano plot displayed the distribution of differentially methylated CpGs with a cut-off value of |Δβ| > 0.3 and *p*-value_BH_ < 0.05 using ggplot2 package [38,39] (v3.4.2). The cut-off score was calculated using the β-value distribution of all samples with mean ± 2SD. Heatmaps were generated using an in-house R script with ComplexHeatmap package [40] (v2.14.0) from individual β-values.

The differentially methylated CpGs result was filtered based on the genomic location, selecting the transcription start site (TSS) regions (TSS200 and TSS1500) and gene body regions using a Δβ cut-off score of >0.3 for hypermethylated and ≤0.3 for hypomethylated. Separated heatmaps were created to visualize the β-value distribution.

For the T1 and T2 comparison, |Δβ| > 0.1 was applied to capture the smaller methylation changes expected in this study. Only 9 individuals had measurements at both T1 and T2, allowing for a valid paired analysis. A power calculation using the observed variability among these fully paired samples showed that this threshold allows detection of CpGs with >96% power (d = 1.4267, n = 9, *p* = 0.05, power = 0.9614).

Enrichment analysis was performed to determine any biological implication of the top 300 differentially methylated genes (based on the highest fold-change) using the Metascape database (http://metascape.org) [41]. We first performed the analysis using all gene regions and then focused on the genomic transcription start site regions TSS1500 and TSS200. Further analysis was performed using the CORONASCAPE database (https://metascape.org/COVID/) [41] to detect similarities and enrichment of differentially methylated genes between our data set and other COVID-19 data sets. Ingenuity Pathway Analysis (IPA) software (version 2023.1) was used to determine potentially activated and inhibited pathway, top regulator analysis, and network summary of the differentially methylated genes in the TSS1500 and TSS200 regions. For the data analysis we used a cutoff ±0.3 alteration levels in the differentially methylated CpG, which gave 34,100 analysis-ready molecules with Δβ spanning from −0.3 to −0.72 and 0.3 to 0.74.

### 2.7. Statistical Analysis

All differences with a *p*-value_BH_  < 0.05 were considered significant, if not stated otherwise. The Bonferroni–Hochberg (BH) correction method was applied. All analyses were performed using R (v4.3.1) with the aforementioned packages.

## 3. Results

### 3.1. Patients and Clinical Characteristics

**Figure 1:**
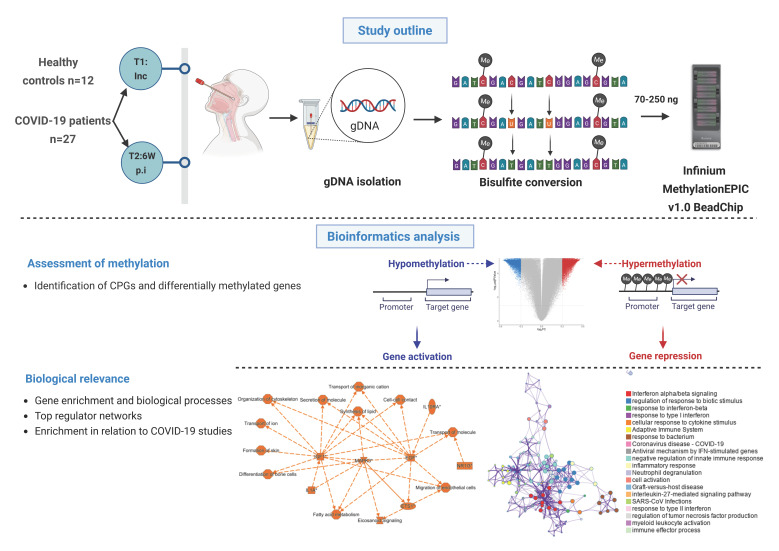
**Graphical summary of the study and methods.** Nasopharyngeal samples were collected from 27 COVID-19 patients at different timepoints, i.e., inclusion (timepoint 1 (T1)); and followed up 6 weeks post-inclusion (T2, 6W post-inclusion), (N = 36). Healthy controls (HCs) (N = 12) were included only at inclusion. Genomic DNA (gDNA) was isolated, bisulfite treated, and methylation analyzed with the Infinium MethylationEPIC v1.0 BeadChip. Bioinformatics analysis was performed to identify unique CpGs and differentially methylated genes, followed by downstream analysis to assess the biological relevance. An asterisk (*) indicates an inferred upstream regulator.

To assess the DNA methylation profiles in the upper respiratory airways of hospitalized COVID-19 patients (N = 27), a total of 36 nasopharyngeal swabs were collected, with 21 samples from hospitalization/inclusion (T1) and 15 samples 6 weeks post-inclusion (T2). In addition, nasopharyngeal swabs from healthy subjects (N = 12) were used as controls (Figure 1). Participants were recruited between July 2020 and October 2021, which represented hospitalized patients with moderate to severe COVID-19 manifestations, as determined by the Guidelines of the National Institutes of Health and with regard to the maximum oxygen required and the highest level of care needed [28]. During this time period the alpha, beta, gamma, and delta SARS-CoV-2 strains circulated in Sweden. Of all patients, 25.9% had at least two of the following underlying conditions, i.e., cardiovascular disease, pulmonary disease, and diabetes mellitus. The median number of days with symptoms prior to inclusion into the study was nine (Table 1). Standard clinical and blood parameters were assessed at inclusion for COVID-19 patients (Table 1) as well as HCs.

### 3.2. Raw DNA Methylation Data Revealed High Quality and Beta Distribution

**Figure 2:**
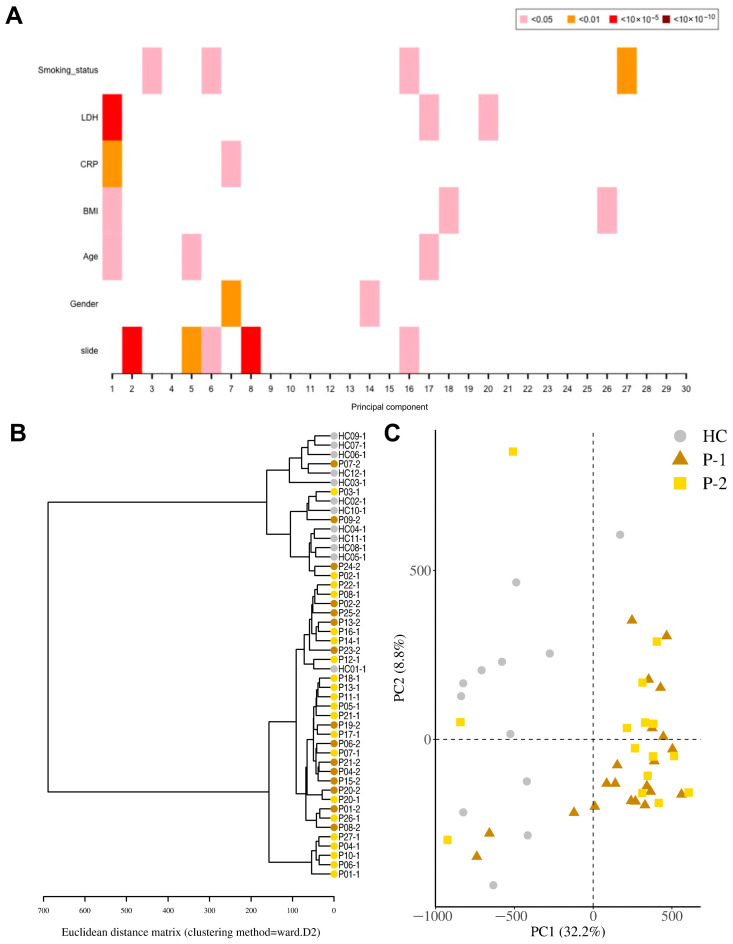
**Distinct separation of COVID-19 patients compared to healthy controls.** Nasopharyngeal samples were collected from 27 COVID-19 patients (P) at inclusion (T1) N = 21 and 6 weeks post-inclusion (T2) N = 15, and from healthy controls (N = 12) (HCs) at inclusion of the study. (**A**) Principal component regression analysis between covariates of interest and the 30 top principal components. (**B**) Hierarchical clustering using the Euclidean distance performed on the β-value data matrix to visualize global correlation was performed. (**C**) Principal component analysis of normalized β-values of COVID-19 patients and healthy controls. Healthy control (HC, circle); COVID-19 patients (Ps) at inclusion or T1 (P-1, triangle) and 6 weeks post-inclusion or T2 (P-2, square).

Genomic DNA (gDNA) from nasopharynx samples were run on a DNA methylation array (Infinium MethylationEPIC v1.0 BeadChip). The data were processed, calibrated, and filtered with bioinformatics analysis to identify methylation patterns and determine their biological significance (Figure 2 and Supplementary Figure S1). Initial quality control assessment of the raw methylation data indicated a good continuous probability distribution, i.e., beta distribution, between samples (Supplementary Figure S1B). LDH and BMI were estimated as significant components of variation/confounding factors in the methylation data set. The PCR analysis (Figure 2A), which quantifies how much of the total methylation variance is explained by each variable, showed that LDH contributed negligibly to this variation (R^2^ < 1 × 10^−5^), so to avoid introducing noise or overfitting it was excluded. This is an approach followed by others in this field [42,43]. Adjusting for disease-associated biomarkers must be performed with caution, as over-adjustment may remove genuine biological signals [44]. Given that LDH is a patient-related factor, this further supports the exclusion and adjustment of the data set for this confounding factor.

### 3.3. DNA Methylation Pattern Among COVID-19 Patients Differs from That of Healthy Controls

For the identification of differentially methylated CpG sites, we assessed the samples from COVID-19 patients, both at inclusion and 6-week timepoints, and HCs. A total of 524,613 statistically significant differentially methylated CpGs were identified in the COVID-19 patient inclusion (T1) and 469,033 in the 6-week timepoint (T2) samples as compared to the HCs. Principal component regression analysis showed univariately associated *p*-values between covariates of interest such as LDH, CRP, and BMI and the 30 top principal components (Figure 2A). To visualize global correlation among T1, T2, and HC samples, we performed hierarchical clustering. Two major clusters emerged, a cluster with controls and a cluster with patients, which indicated a general separation between COVID-19 patients and HCs (Figure 2B). Principal component regression analysis (PCR), an unsupervised learning method, showed a clear separation of hospitalized COVID-19 patients from healthy controls, and revealed the highest variation in the data set (PC1) of 32.2% and the second most variation in (PC2) of 8.8% (Figure 2C). There were exceptions with a few patient samples, which clustered with the healthy controls, as well as one healthy control sample that clustered with COVID-19 patients. The reason for patients clustering with healthy controls could be due to the underlying conditions, which had led to moderate/severe COVID-19 that was not linked to altered airway/lung pathology or medications (pre-COVID-19) that protected the airway compared to the other COVID-19 patients (Table 1). The latter appears to be the case for all patient outliers besides one since they had been on anti-inflammatory medication for chronic pulmonary conditions. Regarding the healthy control individuals that clustered with the patient samples, we could not find an obvious explanation from the clinical data/history.

**Figure 3:**
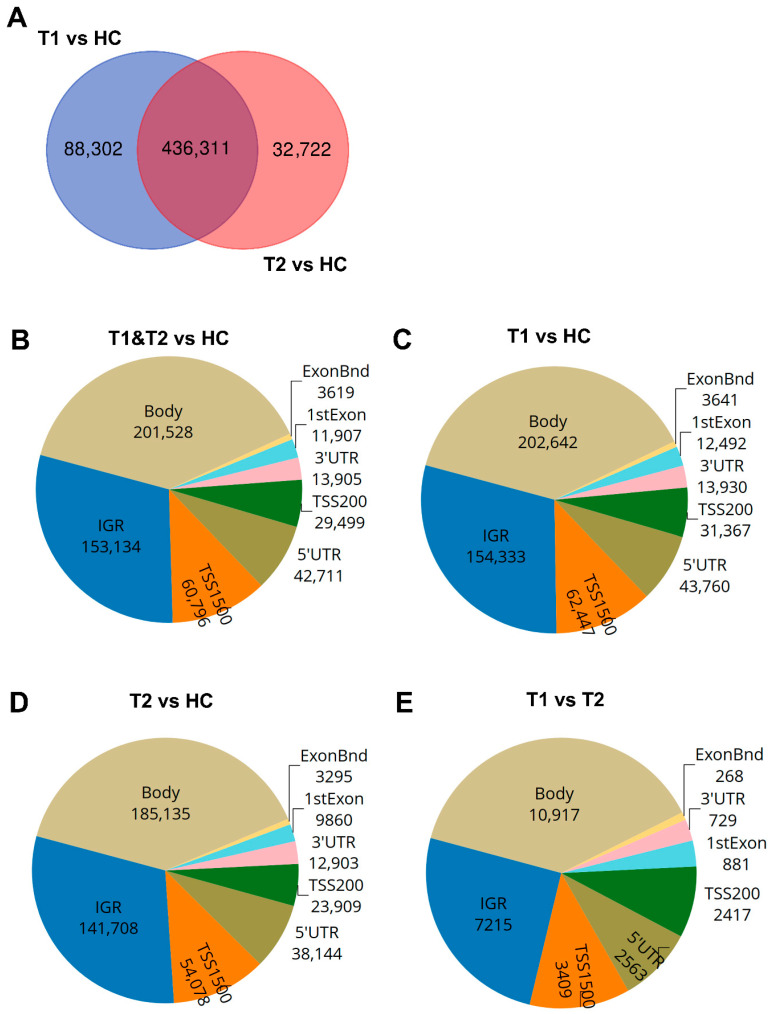
**Distribution of differentially methylated CpGs and genes in COVID-19 patients compared to healthy controls.** Nasopharyngeal samples (N = 36) collected from 27 COVID-19 patients at inclusion (T1) N = 21 and 6 weeks post-inclusion (T2) N = 15 were assessed for differential methylation within the different genomic regions: body, intergenic regions (IGRs), 3′ UTR and 5′ UTR, 1st exon, exon boundaries (ExonBnd), and the transcriptions start sites TSS200 and TSS1500, and compared to healthy controls (HCs) (N = 12). (**A**) Venn diagram of unique and common differentially methylated CpGs. (**B**–**E**) Genomic region distribution of differentially methylated CpGs for COVID-19 patients at T1 and T2 vs. HC, T1 vs. HC, T2 vs. HC, and T1 vs. T2.

The majority of the COVID-19-altered methylated CpG sites remained stable throughout the study, while a subset no longer showed significant group-level differences compared to healthy controls at the 6-week timepoint. Next, we compared the difference and overlap in CpGs between patients and healthy controls as follows: inclusion versus healthy controls (T1 vs. HC), and between the 6-week timepoint versus healthy controls (T2-HC). The T1-HC and T2-HC data sets shared over 400,000 differentially methylated CpGs. However, T1-HC had 88,302 differentially methylated CpGs that were not found in the T2-HC group, and T2-HC had 32,722 differentially methylated CpGs that were not found in the T1-HC (Figure 3A). This indicated that the majority, but not all, of the alterations in CpG methylation remained stable for at least 6 weeks post-COVID-19 (Figure 3A). Based on these findings, we combined the T1 and T2 timepoint samples and compared against healthy control samples to identify the number of significant differentially methylated CpGs in the various genomic regions, i.e., gene body (body), intergenic region (IGR), exon boundaries (ExonBnd), 1st Exon, 3′UTR, 5′UTR, and the transcription start sites (TSSs) 1500 and 200, among COVID-19 patients versus HCs (Figure 3B–E). The greatest number of differentially methylated CpGs in the different genomic regions appeared in the body region (Figure 3B–E).

### 3.4. Differentially Hypomethylated and Hypermethylated Sites/Genes with Lowest p-Values Diverged Between the Inclusion and 6-Week Timepoint in Patients with COVID-19

**Figure 4:**
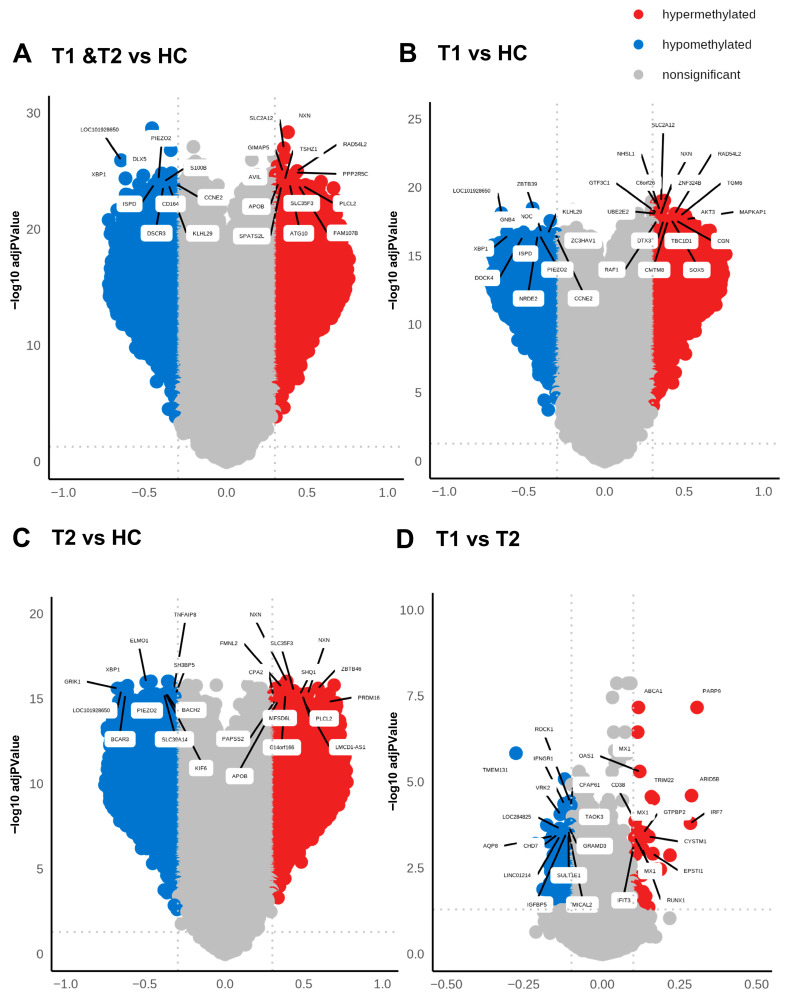
**High level of differentially methylated CpGs in COVID-19 patients compared to healthy controls.** Volcano plots analyzing differentially methylated CpGs **in** all the genomic regions in COVID-19 patients at T1 + T2 vs. HC (**A**), patients at T1 vs. HC (**B**), patients at T2 vs. HC (**C**), and patients at T1 vs. T2 (**D**). The methylation difference (Δβ) with a cut-off > 0.3 for hypermethylated CpGs and cut-off < 0.3 for hypomethylated CpGs and the use of BH-corrected *p*-values < 0.05 cut-off for hypermethylated and hypomethylated CpGs. The top 20 DMGs were annotated using the highest BH-corrected *p*-values for hypermethylated and hypomethylated CpGs.

Methylation can affect gene transcription either by enhancing, decreasing, or silencing transcription [19,20]. Hypomethylated and hypermethylated CpG sites detected in patients with COVID-19 were identified based on Δβ values (Figure 4 and Table 2). The hypermethylated genes with lowest *p*-values for hospitalized COVID-19 patients at inclusion (T1) and at 6 weeks post-inclusion (T2) versus HCs included NXN, SLC2A12, RAD54L2, GIMAP5, and PPP2R5C, while the top five hypomethylated genes with lowest *p*-value were, LOC101928650, DLX5. XBP1, PIEZO2, and S100B **(**Figure 4A). The hypermethylated genes with the lowest *p*-value at T1 encompassed TMEM131, ROCK1, IFNGR1, CFAP61, VRK2, and C6orf138 and the top hypomethylated genes were ZBTB39, PNOC, KLHL29, CCNE2, and ZC3HAV1 (Figure 4B). By 6 weeks post-inclusion (T2), COVID-19 hypermethylation of genes with lowest *p*-value included NXN, FMNL2, ZBTB46, SLC35F3, and SHQ1, and the hypomethylated genes included ELMO1, XBP1, GRIK1, TNFAIP8, and SH3BP5 (Figure 4C). The hypermethylated genes with the lowest *p* values for hospitalized COVID-19 patients at inclusion (T1) vs. at 6 weeks post-inclusion (T2) were PARP9, ABCA1, MX1, OAS1, ARID5B, and the hypomethylated genes included TMEM131, ROCK1, IFNGR1, CFAP61, VRK2, and C6orf138 (Figure 4D). Many of these genes are connected to antiviral and immune responses, and to signaling and inflammation/neuroinflammation.

When exploring the top hypermethylated genes according to the highest fold-change in the patients at inclusion (T1) compared to healthy controls we found TMEM212-AS1, FAM178B, FYN, MIR2117, and TMX2-CTNND1, and among the top hypomethylated genes we found C10orf12, SEMA6D, SFMBT2, PDGFRB, and NISCH. By 6 weeks post-inclusion, COVID-19 hypermethylation of genes with the highest fold-change compared to healthy controls included FAM178B, FYN, TMCC1, TMEM212-AS1, and FIG4, while the top five hypomethylated genes were GIT2, PDGFRB, SEMA6D, TNFAIP8, and ETV6. The top molecules in the COVID-19 T1 and T2 data set with the greatest alteration in methylation compared to healthy controls included hypomethylated MUC20, VAV3, CCL20, and mir21 and hypermethylated GIT2, IFNGR2, FCER1G, and OLR1. Many of these genes with the highest fold-change and lowest *p*-values are related to immune responses and inflammation, and some of them, such as CCL20 and miR-21, have previously been connected with COVID-19 [45,46].

### 3.5. Different Methylated CpGs Patterns Were Evident Among COVID-19 Patients Versus Healthy Controls

**Figure 5:**
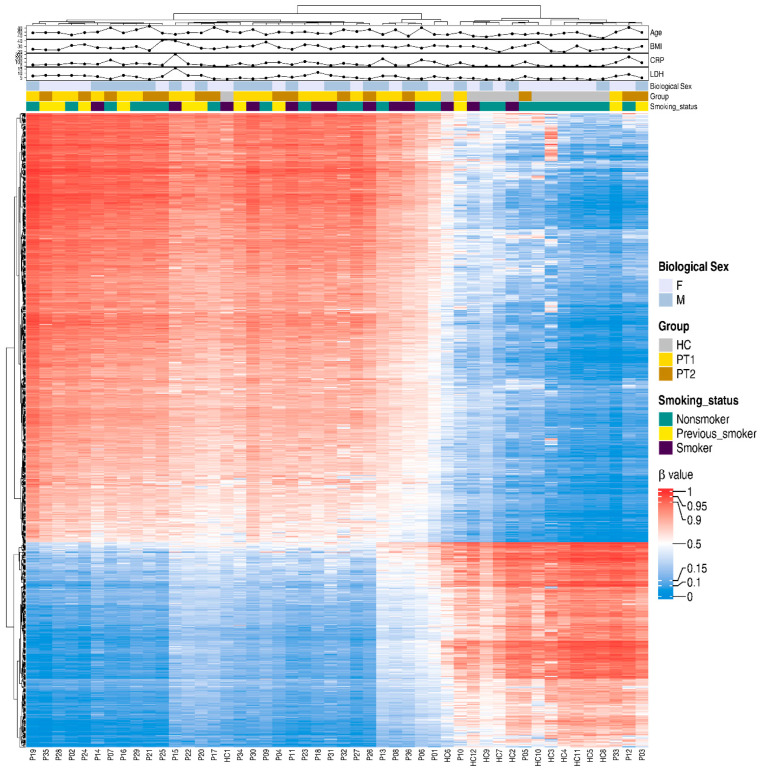
Differentially methylated CpGs between COVID-19 patients and controls in transcription start sites TSS200 and TSS1500. Nasopharyngeal samples (N = 36) collected from 27 COVID-19 patients (P) at inclusion (T1) N = 21 and 6 weeks post-inclusion (T2) N = 15 and healthy controls (N = 12) (HC) were assessed in the TSS200 and TSS1500 regions for the top 1000 hypo- and hypermethylated CpGs, and 40 differentially methylated CpG sites were annotated randomly. Hierarchical clustering against sample groups was performed. Top annotation showed the distribution of sample groups, gender, BMI, age, CRP, smoking, and LDH.

For a snapshot of the overall pattern of differentially methylated CpGs covering all the different genomic regions in hospitalized COVID-19 patients at T1 and T2 versus HCs, a heatmap with hierarchical clustering of the DNA methylation beta-values was constructed with the top 20,000 methylated CpGs. This showed a clear difference in methylation between the COVID-19 patients and controls (Supplementary Figure S2). Following this, we focused on the specific methylation patterns for the promotor region (transcription start sites TSS1500 and TSS200) (Figure 5), as methylations in these areas are good predictors of activation versus silencing of genes [47]. The DNA methylation beta-value was analyzed using hierarchical clustering with the top 1000 differentially methylated CpGs and the distribution of other associated parameters, such as timepoint, age, and biological sex, and clinical parameters associated with disease, i.e., LDH and CRP. There was a distinct pattern of differentially methylated CpGs in the heatmaps of TSS1500 and TSS200 (Figure 5). We also explored methylation patterns in the gene body genomic region, and found similar patterns to those for transcription start sites and when combining all the different genomic regions (Figure 5 and Supplementary Figures S2 and S3). Of note, the COVID-19 patients had, within the selected transcription start sites TSS1500, TSS200, and the gene body, a higher level of methylated CpG sites compared to controls.

### 3.6. Enrichment Analysis of Genes Methylated in the Transcription Start Sites in COVID-19 Patients Indicated a Multilayered Activation of Responses by SARS-CoV-2 Infection

To investigate the biological relevance of methylated genes in COVID-19 patients, we used the top 300 differentially methylated genes with the highest fold-change in an enrichment analysis using Metascape database (http://metascape.org) [48]. Initial analysis of the differentially methylated genes located in the total gene regions from T1 and T2 for all patients versus healthy controls showed the top enriched terms relating to response to stimulus, regulation of biological process, and cellular process (Supplementary Figure S4A), The analysis of genes from all the different genomic regions with differentially methylated CpG between inclusion (T1) and 6 weeks later (T2) in the COVID-19 patients showed an enrichment of ontology clusters relating to response to virus and regulation of neuron projection development (Supplementary Figure S4B). We examined the similarities to other COVID-19 data sets using the CORONASCAPE database (COVID-19 Reference Gene Lists) [48], and several sets matched. Five data sets were selected based on gene overlap. The data sets were from transcriptomic studies on blood and airway samples from SARS-CoV-2-infected patients and in vitro SARS-CoV-2-infected primary human airway epithelial and hypotriploid alveolar basal epithelial cell lines. Across the studies, we found a shared enrichment of genes such as OAS1, OAS3, IFIT3, and MX1, and biological processes relating to antiviral responses, cytokine signaling, interferon signaling, and antiviral mechanism by IFN-stimulated genes, among others (Supplementary Figure S4C).

**Figure 6:**
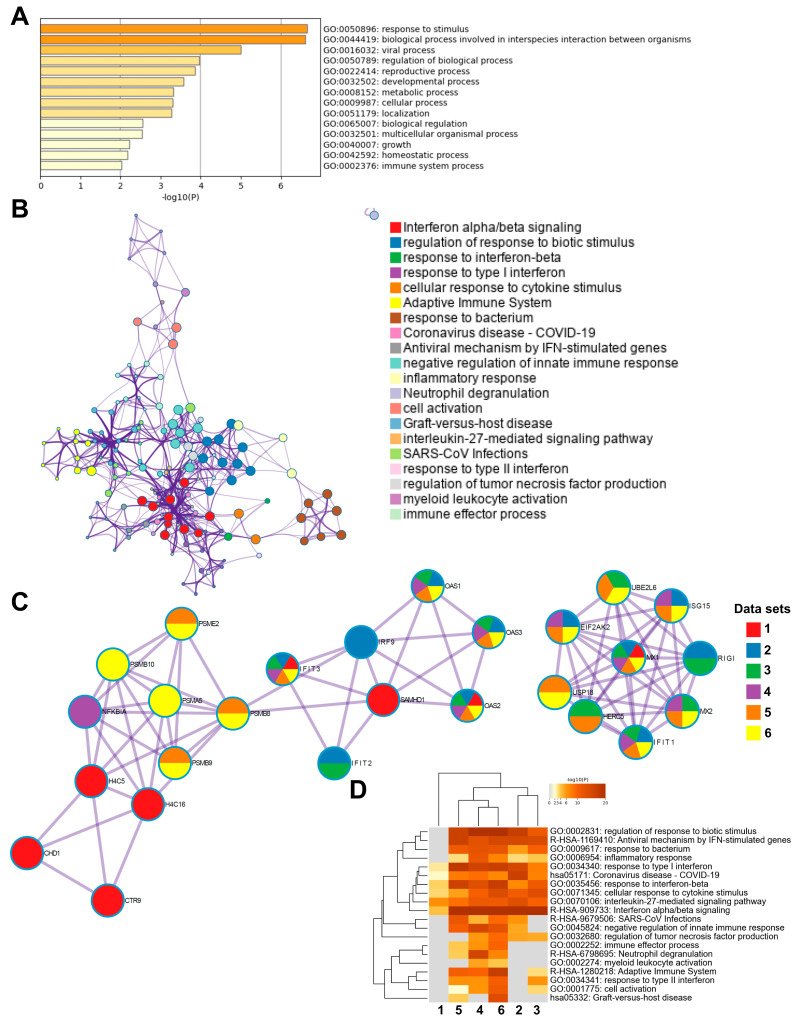
Enrichment analysis of differentially methylated genes and enrichment match analysis for differentially methylated genes among COVID-19-specific studies. Nasopharyngeal samples (N = 36) collected from 27 COVID-19 patients (P) at inclusion (1) N = 21 and 6-weeks post-inclusion (2) N = 15, and from healthy controls (N = 12) (HC), were processed and evaluated for DNA methylation. Analysis was for T1 vs. T2 in the transcription start sites TSS200 and TSS1500 for (**A**) a heatmap of the top-level gene ontology biological process and (**B**) an enriched ontology network for the top 300 differentially methylated genes regarding fold-change. (**C**,**D**) Analysis match performed against the top 5 selected data sets based on overlapping genes from COVID-19 studies (see below) to assess similarities between enrichment of genes. (**C**) Network nodes identifying neighborhoods where genes are densely connected. (**D**) Heatmap of top-level gene ontology biological terms across input gene lists. Each COVID-19 data set has been numbered and color-coded: 1, gene list from the present study; 2, RNA_Blanco-Melo_A549-low-MOI_Up; 3, RNA_Lieberman_Nasopharynx_Infected_vs_Neg_Up; 4, RNA_Wilk_CD14 + Monocytes_patient-C1A-mild_Up; 5, RNA_Zhang_B-cells_severe-and-moderate_Up; 6, RNA_Zhang_NK-cells_severe-and-moderate_Up. Enrichment analysis was performed using Metascape and Coronascape [48].

Next, we performed subsequent enrichment analysis focusing on differentially methylated genes in the TSS regions in the COVID-19 patients at inclusion (T1) versus 6 weeks post-inclusion (T2) (Figure 6). The highest top-level gene ontology biological processes included response to stimulus, biological process involved in interspecies interactions between organisms, and viral process (Figure 6A). There were 20 processes in the enriched ontology network analysis for the TSS regions in our data set including interferon alpha/beta signaling, negative regulation of innate immune response, adaptive immune system, inflammatory response, and antiviral mechanism by IFN-stimulated genes (Figure 6B). The top enriched clusters across SARS-CoV-2/COVID-19 studies included genes OAS1-3, IFIT3, SAMHD1, and MX1 involved in the innate immune response, interferon signaling, and defense response to the virus (Figure 6C). There was some degree of overlap with the findings from this analysis of TSS regions with all genomic regions (Figure 6C and Supplementary Figure S4C). Top enriched terms across COVID-19 studies highlighted interleukin-27-mediated signaling pathway, interferon alpha/beta signaling, and response to interferon-beta as top enriched terms (Figure 6D). Taken together, our findings clearly indicate a multilayered activation of responses by SARS-CoV-2 in the airway in COVID-19 patients at the early timepoint (inclusion/hospitalization).

### 3.7. ERK1/2, IL17A and NOSTRIN Were Identified as Top Regulators, and MAPK3, IGF1, and EGF to Be Involved in Several Biological Processes in COVID-19 Patients

**Figure 7:**
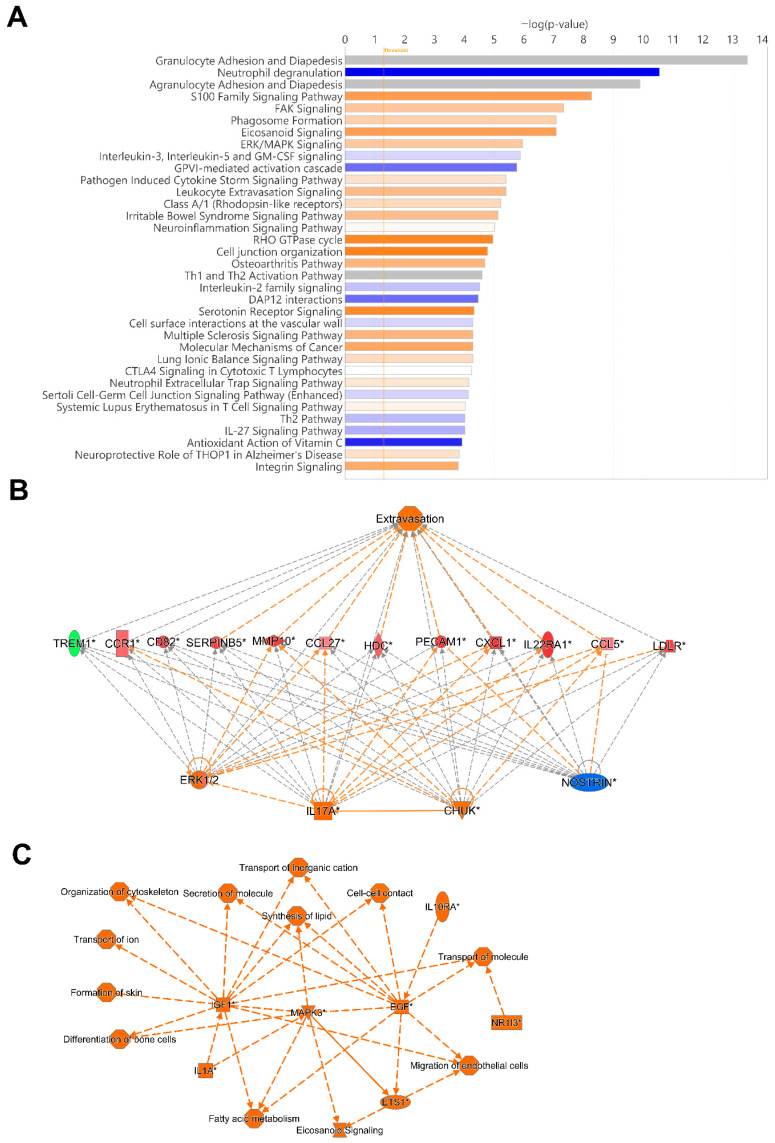
**Top canonical pathway and top regulatory networks in hospitalized COVID-19 patients.** DNA methylation analysis was performed on nasopharyngeal samples from 27 COVID-19 patients (P) at inclusion (1) N = 21 and 6 weeks post-inclusion (2) N = 15, and from healthy controls (N = 12) (HC). (**A**) Canonical pathway analysis was performed with Ingenuity Pathway Analysis to identify the top potentially activated and inhibited pathways linked to the methylated genes located in the transcription start sites TSS1500 and TSS200, with the z-score cut-off set to 1. Positive z-score is indicated in orange, while negative z-score is indicated in blue. Significant TSS differentially methylated CpGs with a cut-off of +/− 0.3-fold-change were assessed in Ingenuity Pathway Analysis for the (**B**) top regulator effect network and for (**C**) graphical summary. An asterisk (*) indicates an inferred upstream regulator.

To identify potentially activated and inhibited canonical pathways, and top regulator networks based on all significant hypomethylated and hypermethylated transcription start site regions encoding genes in our data set, we used Ingenuity Pathway Analysis (IPA) (Figure 7). The top canonical pathways, Granulocyte Adhesion and Diapedesis, and Agranulocyte Adhesion and Diapedesis had a very high significance but no Z-score, i.e., activation or inhibition could not be predicted. Activated canonical pathway included S100 Family Signaling Pathway, FAK Signaling, Phagosome formation, Eicosanoid Signaling, and ERK/MAPK Signaling. The pathways predicted to be inhibited included Neutrophil degranulation, Interleukin-3, Interleukin-5 and GM-CSF signaling, and GVPI-mediated activation cascade (Figure 7A).

The regulators CHUK, IL17A, MAP2K1, ERK1/2, and NOSTRIN were found in a regulator network/pathway with high consistency scores (Figure 7B). NOSTRIN downregulation in COVID-19 reduces nitric oxide production, which could enhance endothelial dysfunction and inflammation. Elevated CHUK (IKK-α) and IL17A drive excessive NF-κB activation and pro-inflammatory cytokines, exacerbating the cytokine storm and ARDS. Additionally, upregulation of MAP2K1/ERK signaling promotes viral replication and inflammation, further increasing disease severity and lung damage (Figure 7B). A summary of the major biological pathways and factors demonstrated the role of MAPK3, IGF1, and EGF as central nodes linked to various processes including tissue repair and activation of immune responses (Figure 7C). Their effects in COVID-19 patients could be part of hyperinflammation for MAPK3, whereas IGF1 and EGF signaling if overactivated it can lead to fibrosis [49,50].

## 4. Discussion

Severe infections as well as different medical conditions alter the DNA methylation pattern in cells and tissues. Here we analyzed nasopharyngeal samples from patients with COVID-19 at inclusion/hospitalization and 6 weeks post-inclusion and from healthy individuals to identify SARS-CoV-2-induced epigenetic signatures by profiling the DNA methylation patterns. We utilized upper respiratory airway samples to explore the effect on the airway as there is evidence that epigenetic alterations that occur in one location or cell type cannot directly be extrapolated for another cell/tissue [51]. There was a clear separation in the DNA methylation pattern in the airway samples between COVID-19 patients and healthy controls, but we could not see a clear distinction in DNA methylation between COVID-19 patients with moderate and severe disease. The high impact exerted by COVID-19 on the DNA methylation pattern in the airway is in line with other studies that explored this in blood samples from COVID-19 patients [15,24,25,26,27,52]. Interestingly, patients who had undergone anti-inflammatory treatments such as oral/nasal inhalation of glucocorticoids for previous/chronic airway manifestations had a different DNA methylation pattern and clustered with healthy controls in the PCA. This suggests that the initial inflammatory response influences the imprinted DNA methylation pattern in the airways and that this did not occur to the same degree among individuals on anti-inflammatory therapy due to less inflammation in the airway. Additionally, pre-COVID-19 treatment in these patients for asthma may have contributed to this effect, as shown by others [53]. Another explanation could be that the anti-inflammatory drugs could have altered the DNA methylation effects exerted by SARS-CoV-2 on the airway and the local infiltration of immune cells.

We observed partial normalization of the epigenetic profile at the group level; although certain COVID-19-associated hypo- and hypermethylated CpGs/genes no longer differed significantly from healthy controls at six weeks, prominent and persistent epigenetic alterations remained. One explanation for the changes in the airway methylation patterns could be altered cell compositions, such as infiltration of immune cells, proliferation of tissue resident cells, and damaged epithelia at the initial phases or during active infection versus a greater healing phase a few weeks after COVID-19 [54,55].

It is evident that some of the genes with differentially methylated CpGs that were present only at inclusion in the airway samples and not at the 6-week post-inclusion timepoint, such as OAS1 and OAS3, were part of the innate type 1 interferon responses to viruses. These genes have been confirmed to be affected in patients with COVID-19 and are linked to clinical outcome [10,25]. In blood samples, others have shown that some DNA methylation alterations are present one year after infection in hospitalized COVID-19 patients [56]. Genome areas with genes and CpGs that returned to normal levels were connected to viral responses such as type I IFN signaling, whereas areas associated with cell activation, leukocyte activation, lymphocyte activation, and immune system remained altered over time [56]. Our findings are in line with previous findings in blood [56] with regard to DNA methylation returning to normal levels in genes involved in viral responses such as the OAS1, whereas genes involved in, e.g., leukocyte activation and immune system, remained altered throughout the study period.

We observed alterations in DNA methylation across all genomic regions in the COVID-19 patients, but focused on the deeper analysis of the DNA hypermethylation and hypomethylation patterns in the transcription promotor regions as this can more easily be translated to silenced and activated genes, respectively [57]. Furthermore, a previous study found differential patterns of COVID-19-induced DNA methylation in blood, primarily in the promoter regions of immune-related genes [27].

The top enriched ontology and biological processes predicted by the pathway analysis in the COVID-19 data set included regulation of cell activation, leukocyte activation, inflammatory responses, leukocyte migration, immune system process, and response to stimuli, which is in accordance with previous COVID-19 studies [25,58]. Our findings clearly indicate a multilayered activation of responses by SARS-CoV-2 early in the infection/at hospitalization.

Signaling pathways predicted to be affected included the Granulocyte Adhesion and Diapedesis, Neutrophil degranulation, Agranulocyte Adhesion and Diapedesis, and FAK Signaling. The pathways Granulocyte Adhesion and Diapedesis and Agranulocyte Adhesion and Diapedesis involve the recruitment of granulocytes and immune cells such as monocytes, T cells, and NK cells to the site of infection/inflammation, which contributes to excessive inflammation including IL17A production and tissue damage [59,60]. In severe COVID-19 cases, hyperactivated neutrophils degranulate excessively, releasing proteases and ROS that damage surrounding tissues, exacerbating lung injury. This pathway also contributes to the formation of neutrophil extracellular traps (NETs), which are implicated in COVID-19-associated coagulopathies [61].

FAK signaling is crucial for maintaining endothelial integrity and responding to stress and this pathway might be involved in the disrupted endothelial barriers and vascular leakages seen in SARS-CoV-2-infected patients [62]. FAK activity is implicated in fibrotic responses seen in post-acute COVID-19 sequelae, particularly in the lungs [63].

NOSTRIN, IL17A, and ERK1/2 were defined as top regulators in our COVID-19 DNA methylation data set. NOSTRIN protein regulates endothelial eNOS activity and NO production, essential for vascular homeostasis, immune modulation, and inflammation control. Lower eNOS activity will worsen endothelial dysfunction, inflammation, and thrombosis. In addition, low NO levels exacerbate the cytokine storm and inflammation and contribute to respiratory failure [64,65]. We found several factors that clearly can be part of driving the inflammatory state in COVID-19 patients, such as CHUK/IKK-α, and IL17A. Upregulation of CHUK/IKK-α activates NF-κB, driving the production of pro-inflammatory cytokines such as IL-6, IL-1β, and TNF-α in SARS-CoV-2 infected patients [66,67]. Elevated IL17A levels have been observed in severe COVID-19, correlating with hyperinflammation and poor prognosis [7,68]. In addition, the MAP2K1 (MEK1) and ERK1/2 signaling pathways were also activated, and they play a pivotal role in cell proliferation and differentiation and in stress responses. SARS-CoV-2 can exploit the MAPK/ERK pathway to enhance viral replication and propagation in the airway [69,70]. In addition, this signaling pathway could contribute to the cytokine storm and systemic inflammation observed in severe COVID-19.

Analysis of the major biological pathways and factors revealed key roles for IGF1, EGF, and MAPK3. IGF1 and EGF support tissue repair after lung injury from SARS-CoV-2, aiding regeneration, but dysregulation of these factors may impair recovery, contributing to fibrosis, particularly in severe COVID-19 or long COVID [49,50]. IGF1 also modulates immune cells, and its alteration during infection may drive immune dysregulation. SARS-CoV-2 activates the MAPK pathway to enhance viral replication [71]. Besides the role in supporting viral replication, MAPK3 hyperactivation can trigger inflammation (e.g., IL-6, TNF-α) and lung epithelial damage, central to severe COVID-19 [72], and today, MAPK pathway inhibitors are under investigation to mitigate inflammation and viral replication [72].

Genes involved in innate immune responses, such as CASP8, INPP5D, ELMO1, FYN, SEMA6D, PDGFRB, MAP3K13, and ADAM10, and genes involved in immune regulation, such as FYN, CASP8, INPP5D, RCAN3, IFNGR2, FCER1G, and MAP3K13, were identified among the differentially methylated genes with the highest fold-change in COVID-19 patients. Some of these genes have been previously connected to COVID-19. The epigenetic imprinting by SARS-CoV-2 infection on these genes involved in innate responses and immune regulations is an important determinate for the disease development [73].

Whilst exploring some of the other genes affected by COVID-19, we also found that the IL-10 gene was hypomethylated in COVID-19 patients at inclusion/hospitalization, indicating an active IL-10 response early on during infection. IL-10 has been shown to play a part in COVID-19 pathogenesis, both during acute infection and post/long COVID-19 [74,75]. Furthermore, we observed alterations of TSSs for genes involved in inflammation, immune and antiviral responses, e.g., TREM1, MMP10, CCL5, HDC, and in transcription, folding, and transcriptional regulation chromatin remodeling, such as TRIM3, KLHL3, CHD1, and H4C5. Infections and inflammation impact many critical steps in the production, control, and modification of new proteins and SARS-CoV-2 has a direct impact on protein translation and protein trafficking [76] and needs post-translational modifications [77]. Exploring matches of our data set with other data sets revealed mutual enrichment of six genes, i.e., OAS1, IFIT3, MX1, SAMHD1.

The enriched top terms defined by the analysis match included regulation of cell activation, leukocyte activation, inflammatory response, regulation of immune effector processes, and neutrophil degranulation. The genes and the biological processes/responses defined in the enrichment analysis are commonly found in numerous COVID-19 studies [78,79,80], reflecting the impact the viral infection and subsequent immune response exert on the host.

We acknowledge that this study has several limitations that may influence the interpretation of our findings. First, while we know which variants were circulating during the study period, the specific SARS-CoV-2 strain infecting each patient was unknown, potentially introducing biological variability. Direct comparative epigenome studies across SARS-CoV-2 strains are currently limited. It has been shown that SARS-CoV-2 proteins, such as ORF8 that can directly perturb chromatin and epigenetic regulation, differ slightly between some SARS-CoV-2 strains [81]. Additionally, different viral variants can cause distinct host transcriptional signatures and can potentially alter the DNA methylation, resulting in unique DNA methylation for the different viral strains [82]. Another limitation regards the patient cohort comorbidities and treatment which could influence airway methylation patterns, as has previously been shown in DNA methylation studies of blood in COVID-19 [53,83]. Severe conditions such as sepsis are connected to changes in DNA methylation patterns in white blood cells in areas with genes involved, e.g., inflammatory pathways and innate and adaptive immune response, which are reflected in altered gene expression [84]. Subsequently, a relevant comparison of our data would be with other severe infectious diseases to determine the DNA methylation alteration that is specific for COVID-19. It is important to also note that the analysis with IPA reflects predicted, but not definitive, activation or inhibition of biological pathways, and these results should be interpreted as hypothesis-generating, which should be further validated through complementary gene expression or proteomic studies. Finally, given the sample size and the limited number of paired samples, the power to assess changes in DNA methylation may be reduced. In addition, a larger cohort could increase the ability to determine with higher precision the unique DNA methylations and patterns for COVID-19.

## 5. Conclusions

In conclusion, to the best of our knowledge this is the first instance of investigation of DNA methylation patterns in the nasal mucosal area of COVID-19 patients. We found a clear distinct DNA methylation pattern in the COVID-19 patients compared to the healthy controls and that most, but not all, altered methylations lasted for more than 6 weeks post-infection. There was an enrichment of genes with altered methylation at transcription start sites associated with inflammation and the immune system in the COVID-19 data set, which might reflect an attempt to dampen inflammation and immune responses.

Our results provide new insight into early immune events in the upper airways during SARS-CoV-2 infection, adding to the current knowledge regarding COVID-19 immunity and providing further links to fully understand this infectious disease and develop improved treatments to reduce the symptoms of infection.

## Figures and Tables

**Table 1:** Clinical and demographical characteristics of hospitalized COVID-19 patients.

Variable	Clinical Data	Reference Range
Number of COVID-19 patients	27	
Age, median (range)	56 (26–91)	
Body mass index, median (range)	28.7 (19.4–41.5)	
Biological sex, % (N)	44.4 F/55.6 M (12 F/15 M)	
Days in hospital, median (range)	6 (2–22)	
ICU/pandemic Ward %, (N)	7.4/92.6 (2/25)	
Days with symptoms before inclusion, median (range)	9 (2–20)	
Spike IgG antibody positive at inclusion, % (N)	63 (17)	
Nucleocapsid IgG antibody positive at inclusion, % (N)	77.8 (21)	
Viral load at inclusion (copies/mL), median (range)	8721.43 (1071.43–3.4 × 10^7^)	
Antiviral treatment, % (N)	22.2 (6)	
Corticosteroid treatment, % (N)	59.3 (16)	
Corticosteroid/Asthma treatment prior to COVID-19, % (N)	14.8 (4)	
No/oxygen/HFNOT:CPAP^1^/mechanical ventilation, % (N)	7.4/44.4/48.1/3.7 (2/12/13/1)	
Cardiovascular disease, % (N)	63 (17)	
Pulmonary disease, % (N)	33.3 (9)	
Diabetes mellitus, % (N)	25.9 (7)	
Two of the underlying conditions, % (N)	25.9 (7)	
Disease score: moderate/severe, % (N)	92.6 (25)/7.4 (2)	
Ongoing smoking/snus, % (N)	0	
Previous history of smoking/snus, % (N)	51.9 (14)	
Leukocytes (×10^9^/L), median (range)	6.7 (2.8–21.2)	3.5–8.8
Thrombocytes (×10^9^/L), median (range)	239 (134–458)	150–400
Lymphocytes (×10^9^/L), median (range)	1.1 (0.4–2.1)	1.1–4.8
Monocytes (×10^9^/L), median (range)	0.4 (0.1–2.24)	0.1–1
Lactate dehydrogenase (µKat/L), median (range)	5.3 (2.5–16)	>70 years < 3.5, <70 years < 4.3
C-reactive protein (mg/L), median (range)	40 (0–318)	0–10

**Table 2:** Top hyper- and hypomethylated genes with unique differentially methylated CpGs in COVID-19 patients compared to controls.

Comparison	Significantly * Hypermethylated Genes	Significantly * Hypomethylated Genes
**COVID-19 patients at inclusion (T1) and 6 weeks post inclusion (T2) vs. HC**	NXN, SLC2A12, RAD54L2, GIMAP5, PPP2R5C, AVIL, TSHZ1, APOB, ATG10, SLC35F3, FAM107B, PLCL2, SPATS2L, CEP350, TNXB, CD2, SIRT2, UGCG	LOC101928650, DLX5. XBP1, PIEZO2, S100B, CCNE2, ISPD, KLHL29, DSCR3, CD164, COL4A1, XBP1, BACH2, CFAP61, ERG, DOCK4, TUBGCP2, GNB4, GPR77
**Patients T1 vs. HC**	NXN, SLC2A12, C6orf26, NHSL1, GTF3C1, RAD54L2, UBE2E2, ZNF324B, AKT3, TGM6, MAPKAP1, TBC1D1, RAF1, DTX3, CGN, CMTM8, SOX5, GIMAP5, SIRT2, EXOSC10	LOC101928650, ZBTB39, PNOC, KLHL29, ZC3HAV1, CCNE2, GNB4, NRDE2, XBP1, PIEZO2, DOCK4, ISPD, B4GALT5, ZNF622, TSS1500, CFAP61, C1orf55, PIGL, DLX5, CLSPN
**Patients T2 vs. HC**	NXN, FMNL2, ZBTB46, SLC35F3, SHQ1, C14orf166, CPA2, PLCL2, PAPSS2, APOB, MFSD6L, PRDM16	ELMO1, XBP1, GRIK1, TNFAIP8, LOC101928650, SH3BP5, BACH2, KIF6, PIEZO2, BCAR3, FYCO1, PIP5K1B,
**Patients T1 vs. T2**	PARP9, ABCA1, MX1, OAS1, ARID5B, TRIM22, CD38, IRF7, GTPBP2, CYSTM1, EPSTI1, AIM2, SERPINA1, DNAJC5B, ZNF337, IFITM1	TMEM131, ROCK1, IFNGR1, CFAP61, VRK2, C6orf138, TAOK3, GRAMD3, LOC284825, MICAL2, IGFBP5, SULT1E1, CHD7, AQP8, LINC01214, TTN-AS1, CUL3, STK39, SREBF2

Δβ cut-off was set to ±0.3 for all groups besides T1 vs. T2 which had ±0.1. * = Significance set as Bonferroni–Hochberg-corrected *p* value < 0.05.

## Data Availability

The original contributions presented in this study are included in the article/Supplementary Materials. Further inquiries can be directed to the corresponding authors.

## Supplementary Materials

The following supporting information can be downloaded at: https://www.mdpi.com/article/10.3390/cells14211673/s1, Figure S1: Quality control to ensure data integrity. (A) Cell-type composition estimation using the normalized data with a reference dataset as provided in the meth_atlas package (see Section 2), (B) β-value distribution of the normalized dataset, (C) quantile-quantile plot (QQplot) for genomic inflation estimation. DNA methylation was assessed in nasopharyngeal samples (N = 36) that were collected from 27 COVID-19 patients (P) at inclusion (T1) and 6 weeks post-inclusion (T2) and from healthy controls (N = 12) (HC) at inclusion of the study; Figure S2: Methylation patterns of COVID-19 patients compared to healthy controls. Nasopharyngeal samples (N = 36) collected from 27 COVID-19 patients (P) at inclusion (1) (N = 21) and 6-weeks post-inclusion (2) (N = 15), and healthy controls (N = 12) (HC) were assessed for differential methylation pattern showing the β-values of the top 20,000 CpGs for all the different genomic regions; Figure S3: The methylation patterns of COVID-19 patients compared to healthy controls in the gene body region. Nasopharyngeal samples (N = 36) collected from 27 COVID-19 patients (P) at inclusion (1) (N = 21) and 6-weeks post-inclusion (2) (N = 15), and healthy controls (N = 12) (HC) were assessed for differential methylation pattern. The heatmap shows the β-values of the top 1000 differentially methylated CpGs in the gene body region; Figure S4. Enrichment analysis and enrichment match analysis for COVID-19-specific differentially methylated genes and enrichment match analysis for top differently methylated genes across COVID-19-specific studies. Nasopharyngeal samples (N = 36) collected from 27 COVID-19 patients (P) at inclusion (1) N = 21 and 6-weeks post-inclusion (2) N = 15, and from healthy controls (N = 12) (HC), were processed and evaluated for DNA methylation. Analysis was performed for (A) enriched terms in T1 and T2 vs HC in all genomic regions (B) biological processes for the top 300 differently methylated genes in T1 vs T2 for all genomic regions. (C) Network nodes identifying neighborhoods where proteins are densely connected. An analysis match for the top 300 differently methylated genes in T1 vs T2 for all genomic regions was performed against the top 5 selected datasets based on overlapping genes from COVID-19 studies to assess similarities between enrichment of genes. Each COVID-19 data set has been numbered and color-coded: 1, gene list from the present study; 2, RNA_Blanco-Melo_A549-low-MOI_Up; 3, RNA_Lieberman_Nasopharynx_Infected_vs_Neg_Up; 4, RNA_Wyler_Calu-3_12h_Up; 5, RNA_Wyler_Calu-3_24h_Up; and 6, RNA_Zhang_B-cells_severe-and-moderate_Up. Enrichment analysis was performed using Metascape and Coronascape [44].
