# Supplementary material for: Altered DNA Methylation Pattern Contributes to Differential Epigenetic Immune Signaling in the Upper Respiratory Airway of Unvaccinated COVID-19 Patients
Source: Cells. 2025 Oct 27;14(21):1673. doi: 10.3390/cells14211673 (PMC12610841; doi:10.3390/cells14211673)
Supplement: Supplementary file 1 [file cells-14-01673-s001.zip › cells-3886875-supplementary.pdf]

Supplementary Figures

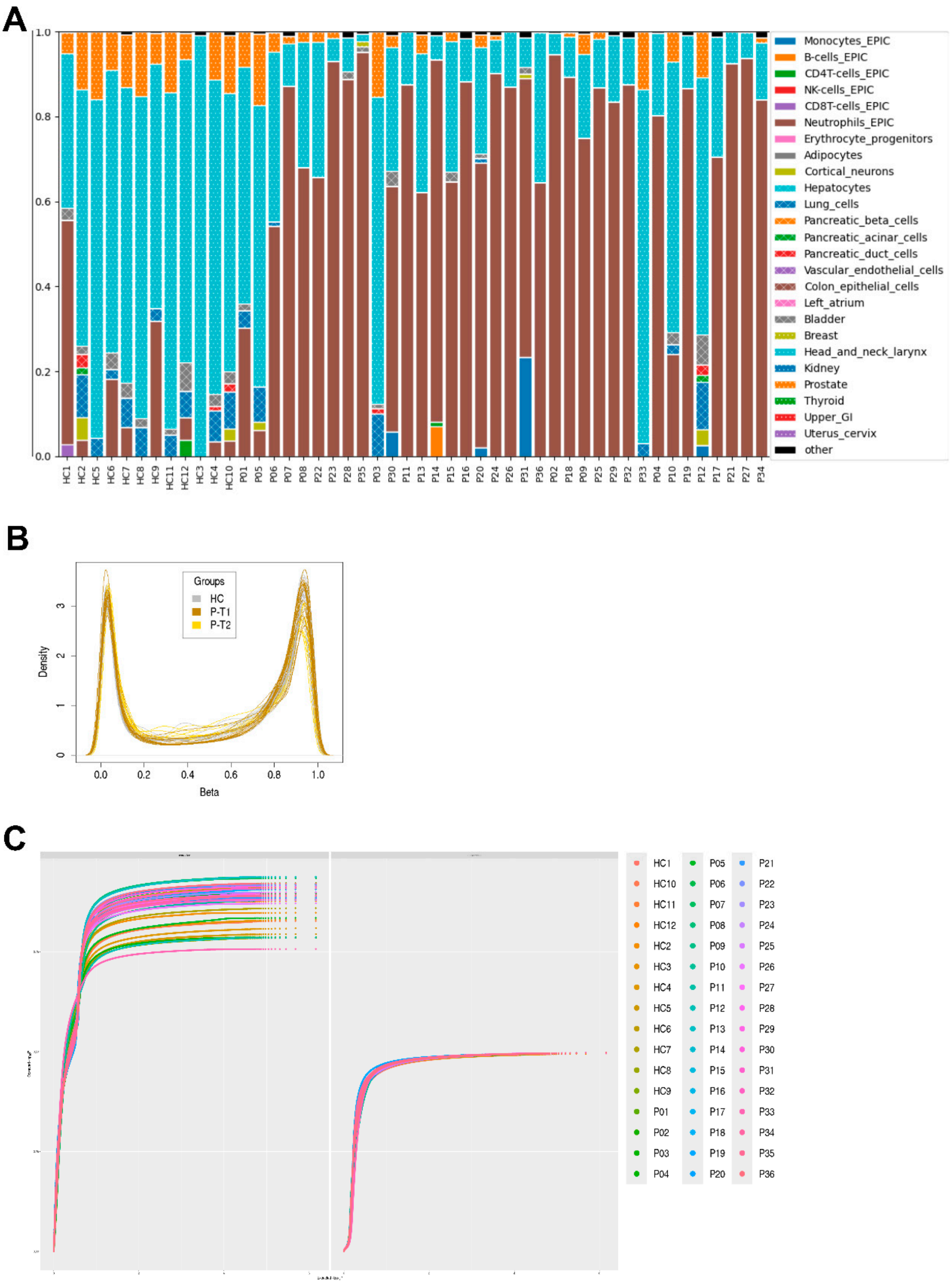

Supplementary Figure S1: Quality control to ensure data integrity. (A) Cell-type composition estimation using the normalized data with a reference dataset as provided in the meth_atlas package (see Section 2), (B) β-value distribution of the normalized dataset, (C) quantile-quantile plot (QQplot) for genomic inflation estimation. DNA methylation was assessed in nasopharyngeal samples (N=36) that were collected from 27 COVID-19 patients (P) at inclusion (T1) and 6 weeks post-inclusion (T2) and from healthy controls (N=12) (HC) at inclusion of the study.

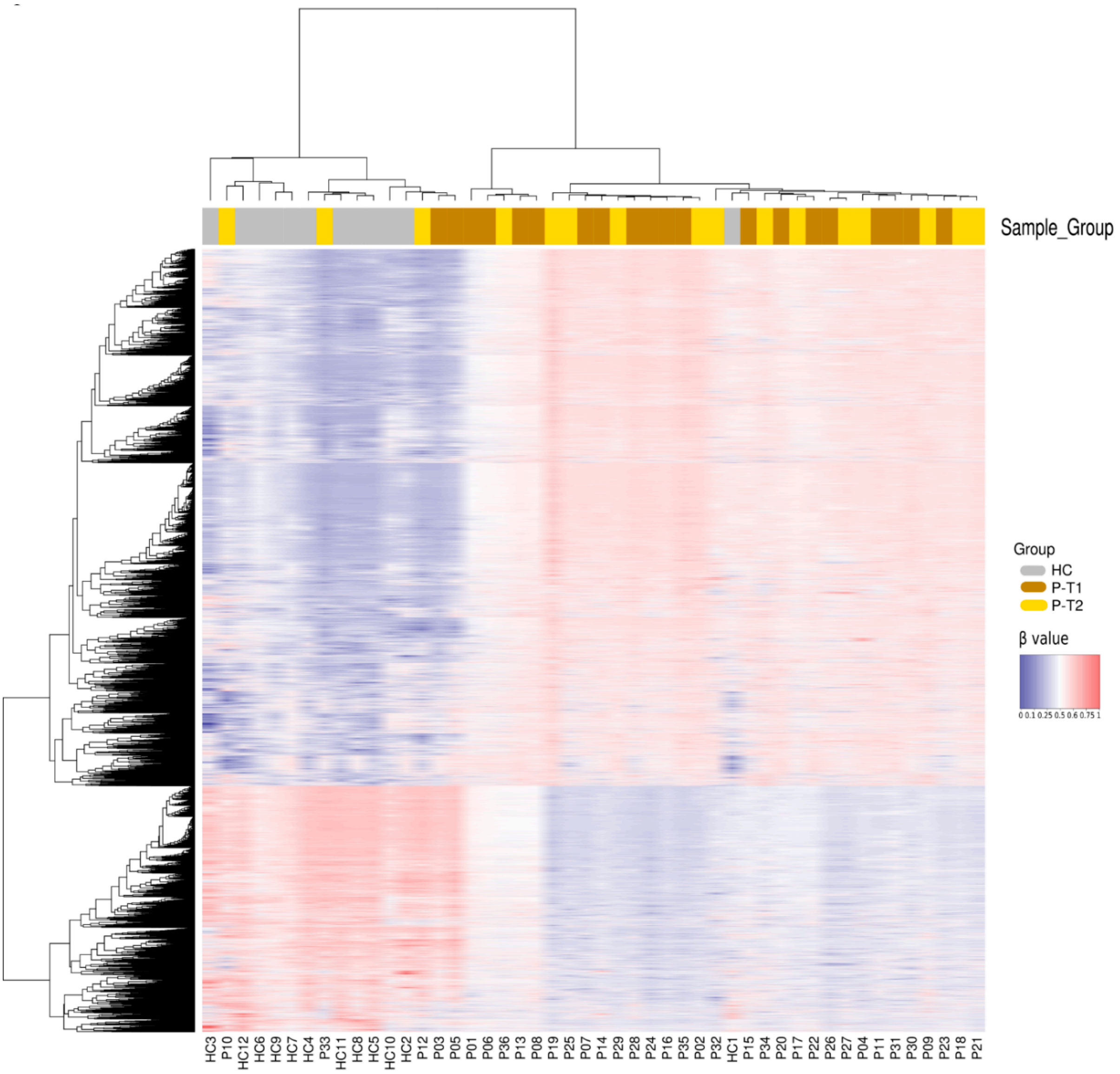

Supplementary Figure S2: Methylation patterns of COVID-19 patients compared to healthy controls. Nasopharyngeal samples (N=36) collected from 27 COVID-19 patients (P) at inclusion (1) (N=21) and 6-weeks post-inclusion (2) (N=15), and healthy controls (N=12) (HC) were assessed for differential methylation pattern showing the **β**-values of the top 20,000 CpGs for all the different genomic regions.

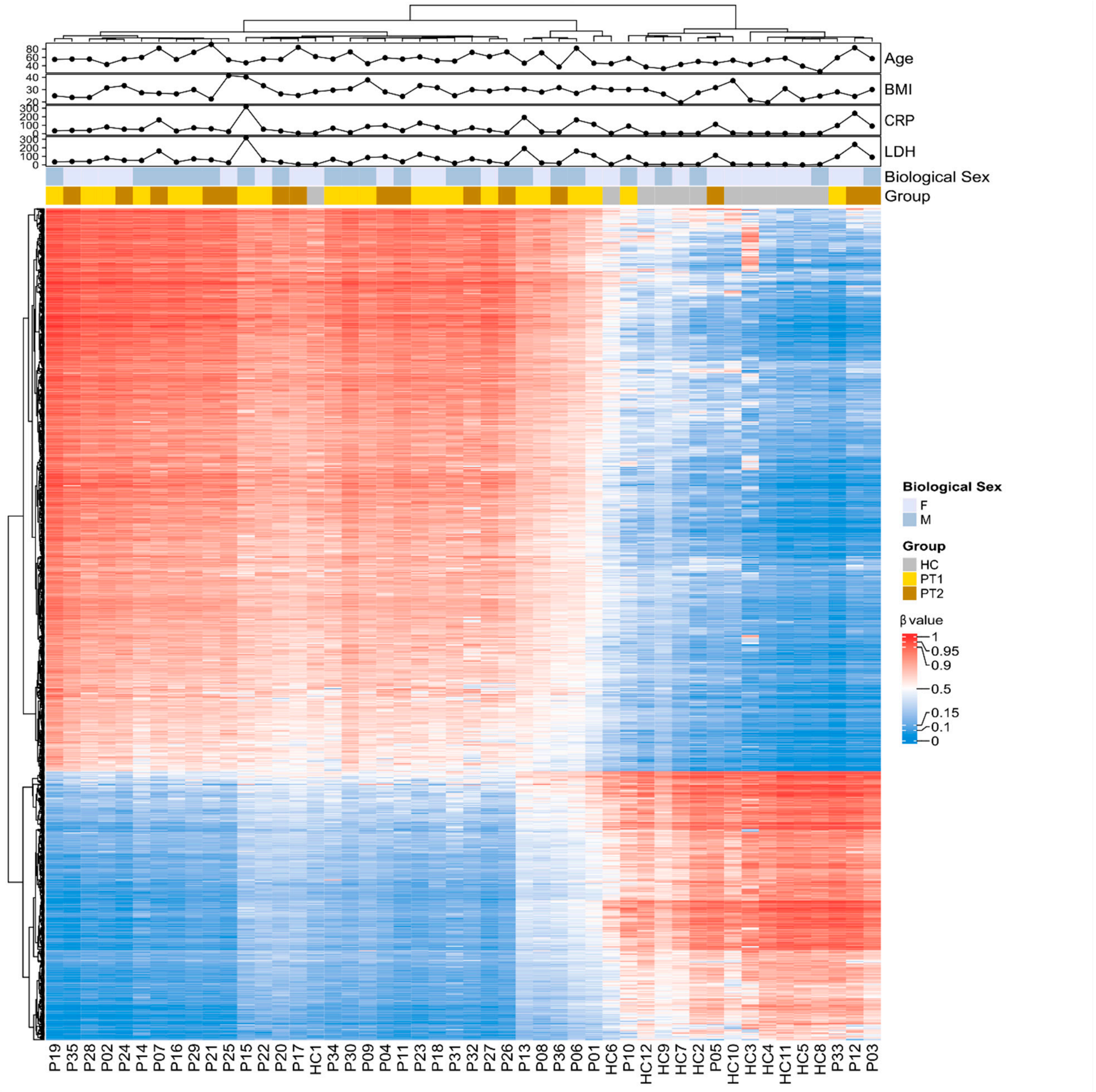

Supplementary Figure S3: The methylation patterns of COVID-19 patients compared to healthy controls in the gene body region. Nasopharyngeal samples (N=36) collected from 27 COVID-19 patients (P) at inclusion (1) (N=21) and 6-weeks post-inclusion (2) (N=15), and healthy controls (N=12) (HC) were assessed for differential methylation pattern. The heatmap shows the **β**-values of the top 1000 differentially methylated CpGs in the gene body region.

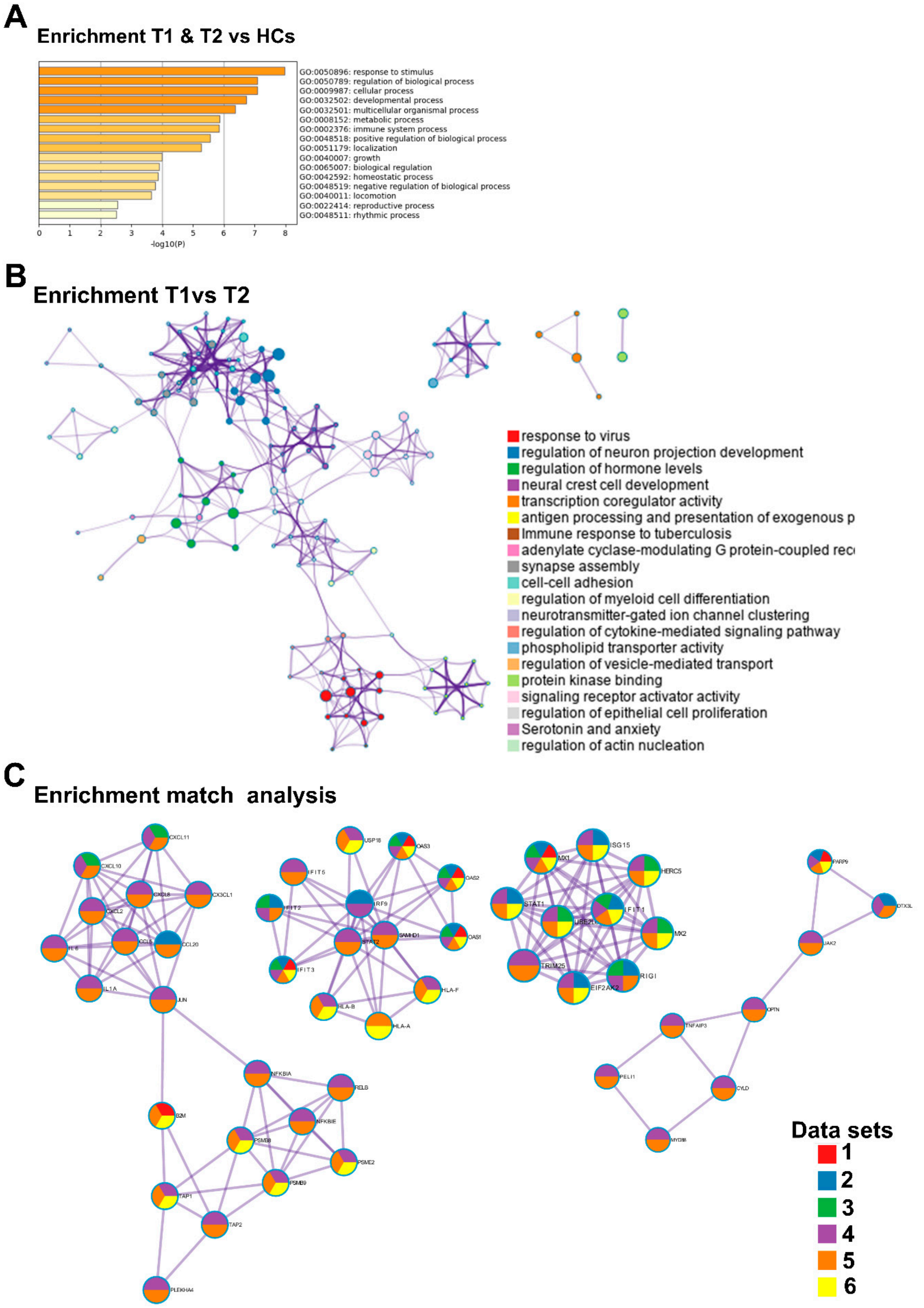

Supplementary Figure S4. Enrichment analysis and enrichment match analysis for COVID-19-specific differentially methylated genes and enrichment match analysis for top differently methylated genes

across COVID-19-specific studies. Nasopharyngeal samples (N=36) collected from 27 COVID-19 patients (P) at inclusion (1) N=21 and 6-weeks post-inclusion (2) N=15, and from healthy controls (N=12) (HC), were processed and evaluated for DNA methylation. Analysis was performed for **(A)** enriched terms in T1 and T2 vs HC in all genomic regions **(B)** biological processes for the top 300 differently methylated genes in T1 vs T2 for all genomic regions. **(C)** Network nodes identifying neighborhoods where proteins are densely connected. An analysis match for the top 300 differently methylated genes in T1 vs T2 for all genomic regions was performed against the top 5 selected datasets based on overlapping genes from COVID-19 studies to assess similarities between enrichment of genes. Each COVID-19 data set has been numbered and color-coded: 1, gene list from the present study; 2, RNA_Blanco-Melo_A549-low-MOI_Up; 3, RNA_Lieberman_Nasopharynx_Infected_vs_Neg_Up; 4, RNA_Wyler_Calu-3_12h_Up; 5, RNA_Wyler_Calu-3_24h_Up; and 6, RNA_Zhang_B-cells_severe-and-moderate_Up. Enrichment analysis was performed using Metascape and Coronascape [44].
